# Approaches and challenges to assessing risk of violence in first episode psychosis: a qualitative interview study of clinicians, patients and carers

**DOI:** 10.1111/eip.13502

**Published:** 2024-02-15

**Authors:** Daniel Whiting, Margaret Glogowska, Seena Fazel, Belinda Lennox

**Affiliations:** a University of Nottingham, Institute of Mental Health; b University of Oxford, Department of Psychiatry; c Oxford Health NHS Foundation Trust; d University of Oxford, Department of Primary Care Health Sciences

**Keywords:** early intervention, violence, offending, risk assessment, qualitative research

## Abstract

**Aim:**

Clinical services for early psychosis seek to improve prognosis for a range of adverse outcomes. For some individuals, perpetration of violence is an important potential outcome to reduce. How these clinical services currently assess this risk however is uncertain. This study aimed to address this gap by using qualitative methods to examine in depth current approaches, attitudes and challenges to assessing violence risk in this clinical setting, from the perspectives of multidisciplinary clinicians, patients and carers.

**Methods:**

Participants were recruited from two UK Early Intervention in Psychosis services. Semi-structured individual interviews were undertaken using a topic guide. In addition, clinical vignettes were presented to clinician participants as a probe to prompt discussion. Data was analysed using thematic analysis, informed by the constant comparative method.

**Results:**

We conducted 30 qualitative interviews, of 18 clinicians and 12 patients and carers. Themes developed from clinician interviews included key difficulties of low confidence, limited training, accessing collateral information and variation in how risk is appraised and communicated. Potential stigma and sensitivity was perceived as a barrier to discussion. Patient and carer perspectives provided insight into how to address barriers, and highlighted the importance of an open approach, including with families.

**Conclusions:**

We conclude with recommendations for developing contextually appropriate pathways to collaboratively assess violence risk and identify modifiable needs to reduce this, and for practical improvements in training and information-sharing.

## Introduction

Schizophrenia spectrum disorders can be associated with a range of adverse outcomes.^1–4^ Clinical services take a prognosis-focussed approach, targeting resources early in illness. Risks and needs are assessed across clinical, social and functional domains. This assessment and care in first episode psychosis (FEP) in many countries is provided by specialist teams, such as Early Intervention in Psychosis (EIP) services in the UK, Australia and Europe^5^ and Coordinated Specialty Care in the US.^6^

For some individuals with schizophrenia spectrum disorders, one potential adverse outcome is violence perpetration. An increased association of violence with psychotic illness is a replicated finding in observational studies,^7^ and consistent with trial evidence that treating psychotic symptoms reduces violence risks.^8^ The first episode of psychosis has been highlighted as a higher-risk phase of illness.^9^ Whilst most individuals who develop a schizophrenia spectrum disorder never behave violently, around 1 in 10 individuals perpetrate violence following first contact with clinical services.^10, 11^ Violence is associated with hospital admission, poorer functional status, and victimisation, ^12–14^ and so the risk of it occurring is directly clinically relevant for services to consider for individual patients, as well as having wider importance from a public health perspective.^15^

Improving violence risk assessment and management in these services, which do not specialise in violence risk, is therefore a clinical priority. However, the manner in which this is currently undertaken, and any associated challenges and needs, has not been described. Studies of risk assessment in general psychiatric services typically focus on risk to self,^16^ which is different in terms of epidemiology, implications, and relation to wider issues such as stigma. How violence is approached clinically is therefore likely influenced by unique issues, particularly in services for FEP with their specific remit.

This study therefore employed qualitative interviews with multidisciplinary EIP clinicians, patients and carers to explore: 1) current approaches, attitudes and challenges to assessing violence risk in FEP, and 2) patient and carer views and experiences of violence risk assessment. Understanding this complex clinical process to inform clinical initiatives required the richness of qualitative methods.^17^

## Methods

### Study design

This was a qualitative study using semi-structured individual interviews.

### Sampling and recruitment: clinicians

Clinicians were recruited from two EIP services in Oxfordshire and Buckinghamshire, UK. Purposive sampling identified relevant groups of multidisciplinary clinicians.^18, 19^ Eligible clinicians were clinically qualified staff with some role in violence risk assessment and management. Due to the covid-19 pandemic, interviews took place via telephone between June 2020 and December 2020. Participants provided informed consent.

### Sampling and recruitment: patients and carers

Patients and carers were recruited from the same two EIP services. All patients had by definition met diagnostic criteria for first episode psychosis as assessed by the Comprehensive Assessment of At-Risk Mental States^20^ as used by the service. Data was analysed from initial interviews from a convenience sample, whereby potential participants were identified by the clinical service, approached and then consented. This was suitable due to the relatively exploratory nature of the work. As categories developed, sampling became more purposive. Including individuals with a history of contact with the justice system was a key characteristic sought during the more purposive phase of sampling, as this was identified as a gap in perspectives during early interviews. In practice however it proved difficult to recruit patients with such histories.

Eligible patients and carers were male or female, aged 14-65 years, able to give informed consent for participation (or parental consent if aged 14-15 years), take part in an interview in English, and deemed suitable to participate by their usual clinician. No selection criteria regarding how long an individual had been known to the clinical service were imposed. The maximum length of time any patient can be under the care of the service is three years. Initial assessments with the service are undertaken within two weeks of referral to the service and are standardised in so far as the same core clinical background history, including a risk assessment, is recorded.

Study interviews occurred via telephone between May 2021 and July 2021. To compensate for their time, patient and carer participants received a £10 online voucher.

### Data collection and analysis

Semi-structured interviews were conducted by DW, capped at 1 hour in length. Topic guides (Appendix 1) were developed based on study aims and discussion within the research team and a public and patient advisory group, and were iteratively adapted as new topics arose. Recruitment continued until sufficient information power was achieved.^21^ For clinician participants only, two fictional clinical vignettes were presented to aid exploration of approaches (Panel 1). See Appendix 2 for details of transcription, thematic analysis,^18^ and how trustworthiness^22^ was addressed, considered across the domains of credibility, transferability, dependability and confirmability and in line with best practice criteria in qualitative research.^23, 24^

## Results

### Clinician interviews

Eighteen clinicians were interviewed (Table 1). Ten hours of dialogue was analysed (average interview length 32 minutes). Data analysis generated three main themes: 1) current practice and focus of violence risk assessment and management, 2) challenges and barriers to assessing violence risk, and 3) general attitudes to violence risk. See Appendix 3 for list of Sub-Themes and exemplar quotes.

### Current practice and focus of violence risk assessment and management

#### a) Depth, structure and timing of violence risk assessment

All clinicians described violence risk as a required, but minor, part of a baseline assessment, and framed it as screening. More in-depth assessment is typically targeted by ‘flags’ of relevance. None considered their approach to be structured, but instead informal information gathering. There was consensus that, after the first assessment, violence risk is not usually reviewed unless indicated, except for acute scenarios.

#### b) Symptom content, context and individuals at risk

Many clinicians focussed on the content of psychotic symptoms. In the vignettes, most clinicians discussed risk to parents in this context. A common view was that fear was a potential trigger for violence, with concerns about associated defensive behaviours including access to weapons. Similarly, several clinicians wanted to explore the content of auditory hallucinations in both vignettes. Several wanted to speak with the parents to understand the triggers and nature of hostility. There was also an emphasis on context and environment in Vignette 2, such as the relevance of unstable accommodation.

#### c) Static and other clinical risk factors

Many clinicians differentiated current risk from static risk factors such as age, sex, or past violence, about which views were mixed. Several stated that the relevance of past violence depended on its context. These mixed views were also apparent in discussion of vignettes. Substance misuse comorbidity was commented on to a variable degree, and less so than some other clinical factors. Family background and early environment was a common focus in Vignette 2. Many thought this was significant in terms of exposure to violence.

#### d) Documenting and describing risk

In terms of the practicalities of recording and communicating risk assessments, most clinicians reported using a risk assessment template with a tick-box for presence of risk, followed by a free-text box. Views on this were mixed. Several felt it was sufficient, by providing information in one location, and allowing clinical narrative. Several however felt its primary function as a list of incidents becomes unhelpful if long or disorganised, without supporting an overall summary. A few described how such documents can be seen as an administrative burden, rather than a meaningful clinical activity.

Clinicians identified no set way that risk is documented or communicated. Typically, clinicians moved away from categorical ratings due to subjectivity, lack of common definition, and a view that this was falsely definitive. A few did describe how, despite reservations, this terminology did still feature in their practice. Others described how they conveyed risk with words such as “significant” or “concerning”.

#### e) Response to violence risk concerns

Most commonly, clinicians described practical steps to maintain the safety of clinicians and families, such as the need for clinicians to visit in pairs, or implications for the sex of the primary worker or setting of contact. These decisions were not standardised.

### Challenges and barriers to assessing violence risk

#### a) Stigma, sensitivity and engagement

Collaborating with patients for violence risk assessment was important to many clinicians. However, there was consensus that the level of patient involvement varied considerably. Concern about stigma was a barrier to clinicians broaching the subject of violence. Further, for those with a history of violence, clinicians were wary of causing distress by enquiring about this, especially during early engagement.

#### b) Lack of established patterns

In these services, individuals are often presenting for the first time. Detailed information on patterns of behaviours was usually absent. Clinicians highlighted how FEP is often evolving and dynamic, and risks could be unknown to patients and their support networks.

#### c) Non-disclosure and access to conviction history

There were reservations about the robustness of information provided by individuals and families about past offending, either because of symptoms, or hesitancy to disclose due to perceived repercussions. Many also highlighted challenges to obtaining formal conviction history, including experiences of clinicians learning this only after caring for an individual for some time. There was uncertainty over the threshold for seeking such information, and how to obtain it.

#### d) Subjectivity of clinical judgement

**Figure 1:**
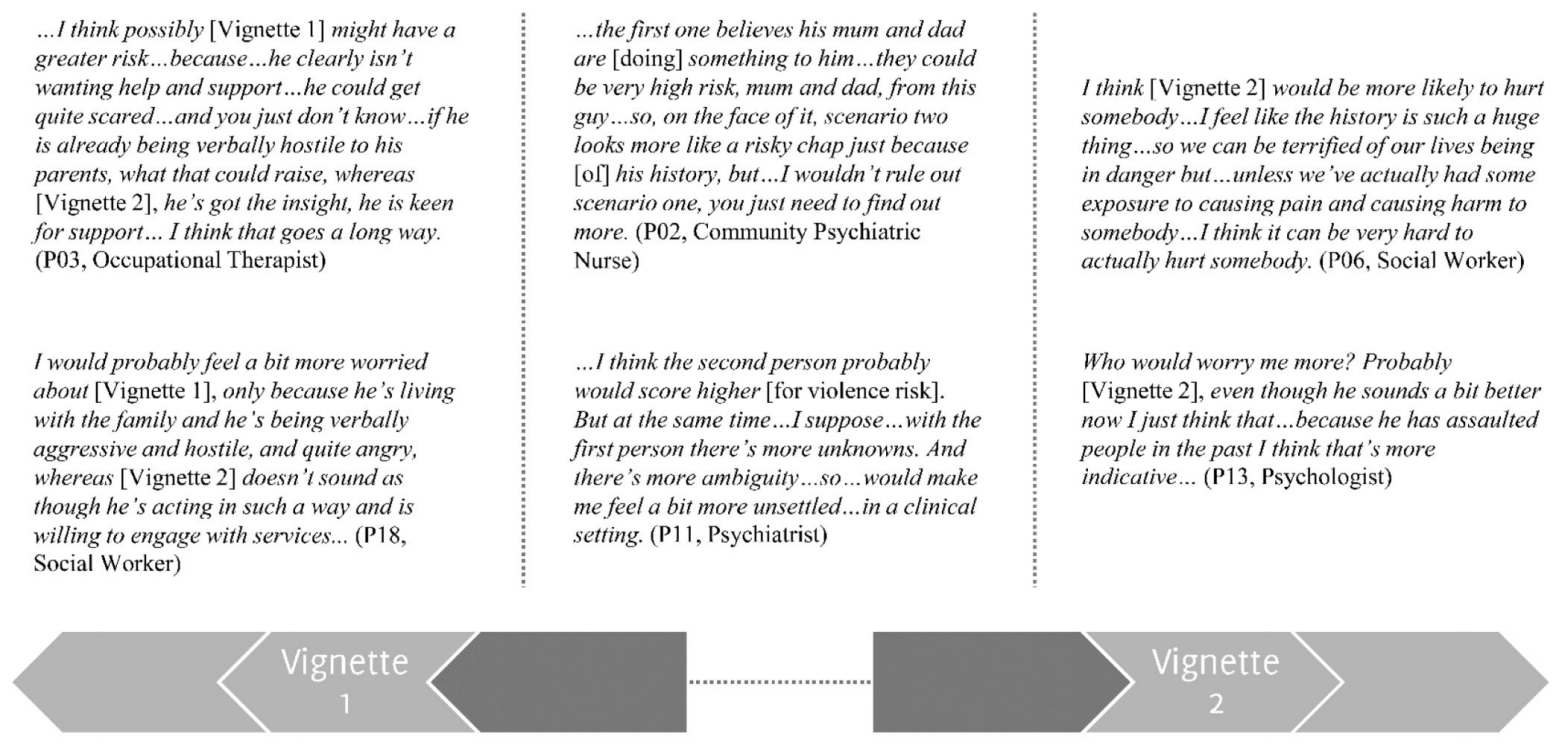
Representative quotes illustrating range of relative concerns regarding violence risk in the two clinical vignettes.

Clinicians found evaluating risk difficult, saying it felt subjective and lacked a formal basis, relying upon pattern-recognition and clinical instinct. This subjectivity was reflected in responses to the clinical vignettes (Figure 1). There was no consistent view on which scenario was more concerning, which varied according to whether more weight was placed on resistance to input from services, or history of violence.

#### e) Handover and inter-agency communication

Many underlined the challenge of handing over risk information, especially when communicating to clinicians outside of the usual care team, or other agencies providing care. Liaising with police was one scenario with potentially different thresholds of concern.

#### f) Time pressure

Many clinicians described struggling to dedicate resources to assess risk meaningfully. Most commonly, clinicians described time pressure, including at a service-level, such as the pressure on inpatient capacity.

### General attitudes to violence risk

Table 2 details the five sub-themes that were developed. There was consensus that violence was clinically relevant, but was not the primary focus of the service. Risk to self was more often considered in practice and training, and clinicians typically lacked confidence in their clinical skills for assessing risk of violence.

### Patient and carer interviews

Twelve patients and carers were interviewed (Table 3). Four hours of interview dialogue was analysed (mean interview length 18 minutes). Data analysis developed three themes, 1) acceptability of broaching topic of violence risk, 2) language and framing of violence risk assessment, and 3) seeking collateral history for risk assessment. See Appendix 4 for themes and exemplar quotes.

#### a) Acceptability of broaching topic of violence risk

There was consensus that violence risk is a legitimate and understandable component of assessment by mental health services. Some framed this in terms of relevance to treatment planning and the safety of staff. A few thought stigma could be an issue. However, this was in terms of media portrayal, and it was stated that it should not prevent health professionals assessing risk. Many did not recall discussion of violence risk being prominent in their assessment. A key aspect of acceptability when it did arise was being part of an interaction that felt professional and supportive.

#### b) Language and framing of violence risk assessment

The use of the word “violence” was discussed. Whilst many agreed that it was a strong word, there was consensus that it best captured the intended meaning. One patient explained that the manner in which it was brought up was more important than words themselves. Many highlighted the importance of being straightforward, and emphasised that it was helpful to make it feel routine. One patient recalled feeling surprised at being asked about criminality amid health-focussed questions. Others agreed that a simple precursor or explanation would help avoid this.

#### c) Seeking collateral history for risk assessment

Patients and carers thought seeking collateral information from police was reasonable where indicated. Some patients thought that individuals with a history of violence might be reluctant to disclose details. One described how they might have avoided this whilst in hospital, as they perceived this would have been linked to more restrictions. A few raised the issue of domestic violence, and that it was important to facilitate families to privately share concerns.

Carers felt strongly that the information they can provide as the people who know the patient best should be integrated into any assessment. This was in the context of all the carers having some experience of feeling not well informed in their family member’s care, including risk issues, due to confidentiality boundaries. Similarly to patients, carers agreed an unambiguous approach to seeking information was preferable.

## Discussion

This study contributes a new, deeper understanding of how violence risk is approached in FEP, and the challenges associated with assessing this in a non-forensic clinical setting, drawing on the experience of clinicians, patients and their carers. It involved 30 interviews with 18 clinicians, and 12 patients and carers, with data analysed thematically informed by the constant comparative method. Three themes and sixteen sub-themes were developed for clinicians, and three themes for patients and carers.

### Current approaches

Clinicians viewed violence as important to consider, with the caveat that risk management is not their services’ primary focus. Typically, assessment of violence risk was a brief screen with fuller exploration only if indicated. Patients reflected this, with few recalling it being prominent during assessment. Patients and carers considered violence risk assessment to be acceptable, relevant to treatment planning and staff safety. When assessed more fully, we found that it was unstructured, based on the clinician’s experience. The content of psychotic symptoms is explored to elicit associated risks, with emphasis on environment and context. Carers highlighted the importance of their involvement, to avoid feeling excluded due to confidentiality barriers.

Approaches to managing violence risk were also explored. Currently, these focus on practical considerations for clinician visits or crisis plans. This mirrors clinical guidelines, which mainly address acute situations.^25^ Some interventions, such as anger management or substance misuse treatment, were mentioned, but with little consensus on their integration.

### Challenges

There were four key areas of difficulty highlighted. First, clinicians’ confidence in their assessment skills and knowledge was low, in part due to violence being overshadowed by risk of suicide and self-harm in both day-to-day focus and training. Training needs in risk assessment have previously been identified in surveys of mental health nurses,^26, 27^ and shown here in strong terms and across disciplines.

Second, clinicians were hesitant to broach the subject of violence for fear of reinforcing stigma, particularly whilst establishing engagement. Concerns about sensitivity were not shared by patients and carers, who did not regard stigma as a reason to avoid discussing risk, and encouraged a straightforward approach. It was suggested that clinicians introduce screening questions in an otherwise health-focussed assessment, and that the enquiry is made to feel routine.

Third, accessing offending history was challenging. Clinicians were wary that this may not be volunteered. Patients pointed out that concerns about repercussions, such as restrictive interventions, might lead to non-disclosure. Despite this, clinicians lacked clarity on justification for seeking collateral information from police, and processes around sourcing this were inconsistent. Patients and carers viewed seeking such information from police as acceptable. Carers also highlighted that intra-familial violence may be challenging to share with clinicians, who should be sensitive to this when establishing family contact.

Fourth, there was inconsistency in how risk factors are evaluated, and how risk is communicated. Inter-clinician variability in weighing-up static risk factors was particularly apparent. Subjectivity was highlighted by clinicians themselves, and by their varied responses to clinical vignettes.

### Implications for practice and research

These findings have implications for clinical services. First, violence risk assessment should receive more emphasis in clinical training. Previous work found that training provision was positively associated with the completeness of psychiatrists’ assessment of risk factors.^28^ Training should also address the need for a straightforward approach to clinical enquiry and carer involvement, and so should involve patients and carers in its development and delivery to ensure acceptability. Further, training should highlight the importance of how violence risk assessment is introduced within a clinical interaction, and that it should be framed explicitly as part of a process of informing care, rather than imposing restrictions. This would reduce concern that patients may feel around disclosure.

Second, clearer processes are needed around proportionate information-sharing between health services and police. Other work has highlighted how triangulation of such information between agencies yields more complete information.^29^ Clinical guidelines for the justification needed to seek such history, and how to broach this transparently with patients, would also be of benefit. There are examples of formal frameworks for inter-agency working around violence that could provide a model for this, such as the Family Violence Multi-Agency Risk Assessment and Management Framework (MARAM) in Australia.^30^

Several areas for improvement were suggested, including consistency of assessments, clinician confidence and communication of risk. The findings highlighted that developments need to ensure that risk is considered proportionately within the remit of the service, without being over-emphasised where it is not relevant.

One way in which other areas of medicine have efficiently translated knowledge about risk factors into clinical practice is the use of simple prediction tools, such as in cardiovascular risk assessment^31^ and cancer medicine,^32^ where these tools are now integral to discussion and personalised treatment planning based on individual risk. In psychiatry, structured tools for violence risk (such as HCR-20) are more typically used as part of the lengthy assessment processes in forensic services. The resource-intensive nature of such tools means that they are not feasible for routine use in non-forensic clinical settings such as EIP services,^33^ and validation studies in relevant populations of people with psychosis are limited.^34^ However, simpler tools now exist that may feasibly, and proportionately, support assessments in non-specialist services, such as OxMIV.^35^ This model weighs 16 routinely available pieces of clinical and sociodemographic information to estimate risk of violence in the 12-months post-assessment, and has been validated in a large clinical cohort of people presenting to UK EIP services.^29^ It performed favourably on measures of net clinical benefit compared with unstructured clinical judgement, and may offer an approach to improve consistency and the low sensitivity of current clinical risk assessments for violence in these settings.^29^ This could enable resources that may reduce risk to be more efficiently allocated to provide maximum benefit. The role and acceptability of such approaches in clinical services for psychosis warrants further exploration.

Finally, the study illustrated the lack of standard approach for addressing modifiable risk factors for violence, with interventions instead focusing on crisis management. Improving this requires a shift away from solely short-term safety planning and towards reducing longitudinal risk. It also requires the development of intervention pathways and psychosocial treatments targeting violence risk.^36^ The findings highlight that such pathways should strive to improve collaboration between clinicians and both patients and carers. Their development should include all these perspectives to ensure that initiatives are feasible and appropriate within the specific clinical context.

### Strengths and limitations

The sample composition, including multidisciplinary clinicians with different background experience and perspectives, as well as patients and carers, was well-placed to understand the phenomenon being examined, and provided rich data and novel insights. By describing the sample and methods and adopting a systematic approach to analysis, findings are anchored in the data. It is possible that a more geographically diverse sample could be considered in future research. However, services engage a defined patient group with a clear model. Developed themes are likely to therefore be transferrable to other such services. Another possible limitation is that those who put themselves forward for participation held stronger views than those who did not participate, and so some of the views expressed may not reflect wider views in the service. Although the patient population included by convenience sampling was broadly representative of the service’s age profile in that most were under the age of 30, a third were aged 41-60 which is a slight overrepresentation. This may be a feature of different profiles of interest and participation in research. Finally, the protocol that received ethical approval allowed only for basic participant information to be recorded, and we did not collect more detailed information such as the diagnostic and clinical profile of participants, which limits the interpretation of what other factors may have contributed to perspectives.

## Conclusion

Violence is an important adverse outcome in clinical services for first episode psychosis. A nuanced approach is required to ensure it is considered in a contextually appropriate manner. This study has highlighted key areas for improvement around training, its framing within a clinical interaction, and information-sharing. To address other challenges such as consistency and clinician confidence, simple structured risk assessment tools should be considered for implementation. These may be developed into pathways that shift focus towards supportive measures to reduce longer-term risk, rather than crisis response alone. Such pathways should ensure contextual acceptability by incorporating perspectives from clinicians, patients and cares in their development, and should seek to facilitate a more collaborative approach to identifying and managing needs around violence risk.

## Supplementary Material

Appendices

## Figures and Tables

**Table 1:** Summary characteristics of included sample. Age bracket rather than calendar age is presented to preserve anonymity of participants.

Participant characteristics		N
Sex	Male	8
	Female	10
Age	21-30	2
	31-40	7
	41-50	3
	>50	6
Professional background	Medical Doctor	4
	Occupational Therapist	2
	Social Worker	4
	Community Psychiatric Nurse	5
	Clinical Psychologist	2
	Other	1
Years working in mental health	0-5	3
	6-10	3
	11-20	6
	>20	6

**Table 2:** Sub-themes developed under main theme of general attitudes to violence risk.

Subtheme	Details
***a)** Clinical importance*	Consensus that violence is clinically relevant. Many stated this was due to specific symptoms, e.g. feeling persecuted and threatened. Others noted the service engages many young men, who are at risk of impulsive behaviour. Comorbidity such as substance misuse was highlighted. A few stated that risk was only relevant for a minority, typically when other factors are present.
***b)** Violence as an “other” risk*	Violence was framed as one of a range of risks assessed, rather than a primary concern. Risk to self was considered higher, and more typically the focus of clinical practice and training. Few received any training specific to violence risk.
***c)** Remit of mental health services*	The appropriate emphasis on violence risk was discussed. One perspective was that it was core due to implications for subsequent clinical contacts. This was balanced by the primary goals being more recovery-focussed. Key was determining whether risk is directly linked to symptoms of mental illness (rather than e.g. personality factors). In the latter, some felt it more a police matter. This was considered difficult to navigate, particularly as risk may have not yet escalated to an offence. Several clinicians however did say that aggression could still be a distinct treatment goal.
***d)** Variations in confidence and experience*	Most clinicians identified violence risk as an area of clinical weakness. They felt they had limited specialist knowledge, and did not encounter violence frequently enough for this to improve. Clinicians were more comfortable monitoring changes in risk with the same patient, or where risk related to treatable symptoms. Personal clinical experience also impacted confidence, such as if clinicians had experienced an assault. Many clinicians stated that there was considerable individual variation in threshold of concern for violence risk, with some noting that risk could become normalised over time.
***e)** Individual clinician culpability and the clinical team*	Several clinicians identified violence risk as a source of anxiety due to accountability should there be a serious untoward incident. Several highlighted the importance of the clinical team for reducing this feeling of personal culpability.

**Table 3:** Patient and carer participant summary characteristics. Age bracket rather than calendar age is presented to preserve anonymity of participants.

Participant ID	Role	Sex	Age bracket
P1	Patient	Female	51-60
P2	Carer (parent)	Female	51-60
P3	Carer (parent)	Female	61-70
P4	Patient	Male	21-30
P5	Patient	Male	51-60
P6	Patient	Female	31-40
P7	Patient	Female	21-30
P8	Patient	Male	21-30
P9	Carer (spouse)	Female	51-60
P10	Patient	Female	21-30
P11	Patient	Male	21-30
P12	Patient	Male	41-50

## Data Availability

The data that support the findings of this study (in the form of exemplar quotes) are available in the supplementary material of this article.

## Panel 1 Fictional clinical vignettes presented to clinician participants during interview.

### Vignette 1

Patient P is a 21-year old man who has just moved back home with his parents after deferring his university degree due to unusually struggling to keep up with work. He has become withdrawn and self-neglecting. He has auditory hallucinations and florid persecutory ideas, including that his parents are feeding information about him to the government. His mother describes him as irritable and verbally hostile and he is angry about being referred to mental health services.

### Vignette 2

Patient M is 25. He is unemployed and currently sofa-surfing. He had a difficult upbringing due to his father being in prison, and was involved in the youth justice system for several years including convictions for assault. He has a long history of self-harm, but now presents with new symptoms of auditory hallucinations in the context of escalating substance use. He has good insight and is keen for support from services.

## References

[1] Hor K, Taylor M (2010). Suicide and schizophrenia: a systematic review of rates and risk factors. J Psychopharmacol.

[2] Fazel S, Wolf A, Palm C, Lichtenstein P (2014). Violent crime, suicide, and premature mortality in patients with schizophrenia and related disorders: a 38-year total population study in Sweden. Lancet Psychiatry.

[3] Sariaslan A, Arseneault L, Larsson H, Lichtenstein P, Fazel S (2020). Risk of subjection to violence and perpetration of violence in persons with psychiatric disorders in Sweden. JAMA Psychiatry.

[4] Immonen J, Jääskeläinen E, Korpela H, Miettunen J (2017). Age at onset and the outcomes of schizophrenia: a systematic review and meta-analysis. Early Interv Psychiatry.

[5] Puntis S, Minichino A, De Crescenzo F, Harrison R, Cipriani A, Lennox B (2020). Specialised early intervention teams for recent-onset psychosis. Cochrane Database of Syst Rev.

[6] Murphy SM, Kucukgoncu S, Bao Y, Li F, Tek C, Breitborde NJK (2018). An economic evaluation of coordinated specialty care (CSC) services for first-episode psychosis in the U.S. public sector. J Psychiatr Ment Health Nurs.

[7] Whiting D, Gulati G, Geddes JR, Fazel S (2022). Association of schizophrenia spectrum disorders and violence perpetration in adults and adolescents from 15 countries: a systematic review and meta-analysis. JAMA Psychiatry.

[8] Ceraso A, Lin JJ, Schneider-Thoma J, Siafis S, Tardy M, Komossa K (2020). Maintenance treatment with antipsychotic drugs for schizophrenia. Cochrane Database Syst Rev.

[9] Nielssen O, Lin Yee N, Millard M, Large M (2011). Comparison of first-episode and previously treated persons with psychosis found NGMI for a violent offense. Psychiatr Serv.

[10] Whiting D, Lennox BR, Fazel S (2020). Violent outcomes in first-episode psychosis: A clinical cohort study. Early Interv Psychiatry.

[11] Winsper C, Ganapathy R, Marwaha S, Large M, Birchwood M, Singh SP (2013). A systematic review and meta-regression analysis of aggression during the first episode of psychosis. Acta Psychiatr Scand.

[12] Cotton SM, Lambert M, Schimmelmann BG, Filia K, Rayner V, Hides L (2017). Predictors of functional status at service entry and discharge among young people with first episode psychosis. Soc Psychiatry Psychiatric Epidemiol.

[13] Hachtel H, Harries C, Luebbers S, Ogloff JR (2018). Violent offending in schizophrenia spectrum disorders preceding and following diagnosis. Aust N Z J Psychiatry.

[14] Shrivastava A, Shah N, Johnston M, Stitt L, Thakar M (2010). Predictors of long-term outcome of first-episode schizophrenia: a ten-year follow-up study. Indian J Psychiatry.

[15] Senior M, Fazel S, Tsiachristas A (2020). The economic impact of violence perpetration in severe mental illness: a retrospective, prevalence-based analysis in England and Wales. The Lancet Public Health.

[16] Graney J, Hunt IM, Quinlivan L, Rodway C, Turnbull P, Gianatsi M (2020). Suicide risk assessment in UK mental health services: a national mixed-methods study. Lancet Psychiatry.

[17] Sofaer S (1999). Qualitative methods: what are they and why use them?. Health Serv Res.

[18] Ritchie J, Lewis J, Nicholls CM, Ormston R (2013). Qualitative research practice: a guide for social science students and researchers.

[19] Palinkas LA, Horwitz SM, Green CA, Wisdom JP, Duan N, Hoagwood K (2015). Purposeful sampling for qualitative data collection and analysis in mixed method implementation research. Adm Policy Ment Health.

[20] Yung AR, Yung AR, Yuen Pan, Mcgorry PD, Phillips LJ, Kelly D (2005). Mapping the onset of psychosis: the comprehensive assessment of at-risk mental states. Aust N Z J Psychiatry.

[21] Malterud K, Siersma VD, Guassora AD (2016). Sample size in qualitative interview studies: guided by information power. Qual Health Res.

[22] Lincoln Y, G EG (1985). Naturalistic inquiry.

[23] Malterud K (2001). Qualitative research: standards, challenges, and guidelines. Lancet.

[24] Tong A, Sainsbury P, Craig J (2007). Consolidated criteria for reporting qualitative research (COREQ): a 32-item checklist for interviews and focus groups. Int J Qual Health Care.

[25] Whiting D, Lichtenstein P, Fazel S (2021). Violence and mental disorders: a structured review of associations by individual diagnoses, risk factors, and risk assessment. Lancet Psychiatry.

[26] Higgins A, Doyle L, Downes C, Morrissey J, Costello P, Brennan M (2016). There is more to risk and safety planning than dramatic risks: mental health nurses' risk assessment and safety-management practice. Int J Ment Health Nurs.

[27] Murphy N (2004). An investigation into how community mental health nurses assess the risk of violence from their clients. J Psychiatr Ment Health Nurs.

[28] Wong L, Morgan A, Wilkie T, Barbaree H (2012). Quality of resident violence risk assessments in psychiatric emergency settings. Can J Psychiatry.

[29] Whiting D, Mallett S, Lennox B, Fazel S (2023). Assessing violence risk in first-episode psychosis: external validation, updating and net benefit of a prediction tool (OxMIV). BMJ Ment Health.

[30] Victoria State Government (2018). Family violence multi-agency risk assessment and management framework: a shared responsibility for assessing and managing family violence risk.

[31] Hippisley-Cox J, Coupland C, Brindle P (2017). Development and validation of QRISK3 risk prediction algorithms to estimate future risk of cardiovascular disease: prospective cohort study. BMJ.

[32] Candido Dos Reis FJ, Wishart GC, Dicks EM, Greenberg D, Rashbass J, Schmidt MK (2017). An updated PREDICT breast cancer prognostication and treatment benefit prediction model with independent validation. Breast Cancer Res.

[33] Viljoen JL, McLachlan K, Vincent GM (2010). Assessing violence risk and psychopathy in juvenile and adult offenders: a survey of clinical practices. Assessment.

[34] Singh JP, Serper M, Reinharth J, Fazel S (2011). Structured assessment of violence risk in schizophrenia and other psychiatric disorders: a systematic review of the validity, reliability, and item content of 10 available instruments. Schizophr Bull.

[35] Fazel S, Wolf A, Larsson H, Lichtenstein P, Mallett S, Fanshawe TR (2017). Identification of low risk of violent crime in severe mental illness with a clinical prediction tool (Oxford Mental Illness and Violence tool [OxMIV]): a derivation and validation study. Lancet Psychiatry.

[36] Khan A, Lindenmayer J-P, Insel B, Seddo M, Demirli E, DeFazio K (2022). Computerized cognitive and social cognition training in schizophrenia for impulsive aggression. Schizophr Res.

