# Supplementary material for: Approaches and challenges to assessing risk of violence in first episode psychosis: a qualitative interview study of clinicians, patients and carers
Source: Early Interv Psychiatry. Author manuscript; Available in PMC 2025 Sep 2. (PMC7618059; doi:10.1111/eip.13502)
Supplement: Appendices [file EMS208250-supplement-Appendices.docx]

**Approaches and challenges to assessing risk of violence in first episode psychosis: a qualitative interview study of clinicians, patients and carers**

Daniel Whiting, Margaret Glogowska, Seena Fazel and Belinda Lennox

**Supplementary Material**

**Appendix 1**

Topic guides for interviews with patients, carers and clinicians (note within interviews discrete topics not covered in current study).

*Clinician interviews*

General questions about violence risk assessment

- Do you routinely assess violence risk?
- How important do you feel this is in your service?
- How do you assess violence risk?
- How much do you involve patients in assessing violence risk?
- How do you communicate your assessment to patients? (probe whether discussed or recorded in letters / CPA documents)
- How confident are you at assessing risk of violence?
- Have you received any training on how to assess violence risk?
- What are the main difficulties with assessing violence risk in your setting?

Questions about short clinical vignettes

- How would you rate the risk of violence in each case?
- What factors are you mainly basing your views on?
- What other things you would you most want to know in each case?

Questions about structured risk assessment tools

- Have you ever used some kind of tool to structure your violence risk assessments?
- Do you think having a structured tool would be helpful?
- What would make you want to use a tool? (probe different properties)
- What would make you not want to use a tool?
- What kind of % risk of violence would reassure, or worry you?
- Do you have any other thoughts or reflections about anything we have discussed?

*Patient interviews*

- Can you tell me about a recent appointment in which risk to others or risk of aggression/violence was talked about, or mentioned in a subsequent letter or careplan? (remind that whether risk was increased or low is not relevant)
- How did you feel about this risk being mentioned, even if it was to say it was low? (probe issues such as labelling and stigma)
- Do you remember an assessment tool called OxMIV being mentioned?
- What did your clinician tell you about why OxMIV was being used?
- What did you think about a tool being used to help? Did it feel like something that was designed to help you as well as clinicians?
- Were you told what the clinician thought about your risk? Did you agree with this or was it a surprise to you?
- Did this lead to a wider discussion of your needs?
- Do you have a sense of whether the tool helped the clinician decide what to do next or what to discuss with you?
- Were you worried that the assessment might lead to something unhelpful for you?
- Do you feel you were as involved in the process of assessing risk as you wanted to be?
- Do you have other good or bad experiences of clinicians assessing risk?
- What advice would you give to clinicians about assessing and discussing risk of violence?
- Do you have any other thoughts or reflections?

*Carer interviews*

- Can you tell me about a recent appointment in which risk to others or risk of aggression/violence was talked about, or mentioned in a subsequent letter or careplan? (remind that whether risk was increased or low is not relevant)
- How did you feel about this risk being mentioned, even if it was to say it was low? (probe issues such as labelling and stigma)
- Do you remember an assessment tool called OxMIV being mentioned?
- What did the clinician tell you about why OxMIV was being used?
- What did you think about a tool being used to help? Did it feel like something that was designed to help you and the person you care for as well as clinicians?
- Were you told what the clinician thought about risk? Did you agree with this or was it a surprise to you?
- Did this lead to a wider discussion of needs for the person you care for?
- Do you have a sense of whether the tool helped the clinician decide what to do next or what to discuss with you and/or the person you care for?
- Were you worried that the assessment might lead to something unhelpful?
- Do you feel you were as involved in the process of assessing risk as you wanted to be?
- Do you have other good or bad experiences of clinicians assessing risk?
- What advice would you give to clinicians about assessing and discussing risk of violence?
- Do you have any other thoughts or reflections?

**Appendix 2**

### Transcription

Interviews were recorded using an encrypted digital voice recorder and transcribed verbatim by a professional service. A service agreement including data protection arrangements was in place. Any potentially identifying information was removed from transcripts. Recordings were destroyed after transcription.

### Data Analysis

Transcripts were imported into NVivo 12^1^ to support a systematic approach to data management and analysis. Data was analysed thematically by DW, with developing themes checked and shared with the research team (who are from different clinical backgrounds and so will have different perspectives), as is standard practice in qualitative research.^2^ Analysis was informed by the constant comparative method^3^ whereby collected data iteratively informed ongoing data collection. Idea-by-idea open coding was built into wider categories which were refined as data were added, and developed into overall themes. Themes developed from clinician interviews, such as stigma, were built into patient and carer topic guides.

### Trustworthiness

Best practice criteria in qualitative research were considered throughout.^4-6^ Trustworthiness^7^ was addressed by ensuring that analysis was systematic, transparent and comprehensive, with accurate recording of the data and justification for decisions made in analysis. Memos and notes were used during the initial stages of analysis to document an “audit trail” of decisions as coding progressed through to interpretation. Findings are therefore confirmable and demonstrably based upon the data. Clear reporting of the sampling strategy, participants and setting allows judgements around transferability to be made. Triangulation (both between cases and sources of data) adds further to the credibility, and there was “fair-dealing”, whereby the design of the studies specifically incorporated a wide range of perspectives.

There was an explicit awareness of how a researcher’s background and motives may shape data collection and analysis.^5^ Specifically, DW is a forensic psychiatrist with an interest in improving approaches to risk assessment. The process above was to mitigate the risk of this impacting results.

1. QSR International Pty Ltd. (2020) NVivo
2. Ritchie J, Lewis J, Nicholls CM, Ormston R. Qualitative research practice: a guide for social science students and researchers. Sage. 2013.
3. Boeije H. A purposeful approach to the constant comparative method in the analysis of qualitative interviews. *Qual Quant.* 2002;36(4):391-409.
4. Giacomini MK, Cook DJ. Users' guides to the medical literature: XXIII. qualitative research in health care A. Are the results of the study valid? *JAMA.* 2000;284(3):357-362.
5. Malterud K. Qualitative research: standards, challenges, and guidelines. *Lancet.* 2001;358(9280):483-488.
6. Tong A, Sainsbury P, Craig J. Consolidated criteria for reporting qualitative research (COREQ): a 32-item checklist for interviews and focus groups. *Int J Qual Health Care.* 2007;19(6):349-357.
7. Lincoln YG, EG. Naturalistic Inquiry. Sage Publications. 1985.

**Appendix 3**

Table of sub-themes and exemplar quotes from clinician interviews.

| **Themes** | **Sub-Themes** | **Exemplar Quotes** |
| --- | --- | --- |
| Current practice and focus of violence risk assessment and management | 1. Depth, structure and timing of violence risk assessment | *…I don’t [have a uniform approach]. I just have a conversation…considering what’s in the previous notes, or the GP referral, and a bit of discussion with them…I don’t have any sort of set form or set questions I ask really.* (P08, Social Worker)  *…if somebody’s known to be someone who carries a knife…I would always…check…those things with them…It’s ongoing for people where it seems to need to be ongoing.* (P15, Psychiatrist) |
|  | 1. Symptom content, context and individuals at risk | *…he’s thinking that the parents are feeding information to the government and that makes me feel* [there is a] *risk that he might try to take measures to stop them.* (P13, Psychologist) [Vignette 1]  *…I’d be wondering…what the degree was and to what extent he’s feeling, for example, like, he might have to defend himself. Whether he’s needing to have…a knife by his bed or how safe he feels with his parents and whether he’s an ongoing risk to his parents.* (P06, Social Worker) [Vignette 1] |
|  | 1. Static and other clinical risk factors | *….he had violent offences…when he was in the youth offending team…that is now nine years ago. So things may have…been completely fine. You don’t know, do you? I’d probably go on asking him sort of has he been in trouble with the police since…I suppose I wouldn’t necessarily draw myself straight to the fact that he’s had a number of sentences without maybe considering the context of those a bit more.* (P08, Social Worker) [Vignette 2]  *…I’d like to like learn from him about what the context was of the assaults in the past, but I wouldn’t…dig into them if it seemed quite irrelevant to the current situation. But if it was…the same triggers that he’s still dealing with now, then I would think he maybe would want some support in how to avoid things turning violent.* (P12, Community Psychiatric Nurse) [Vignette 2]  *…the thing that I’m aware of with…violence risk assessment…is that actually…we don’t pay enough attention to static risk factors. We pay more attention to dynamic risk factors and so some of the static ones can get missed…like being a young male with a previous criminal record.* (P01, Psychiatrist) |
|  | 1. Documenting and describing risk | *Oh, it is difficult…with the low/medium/high…What on earth do you mean by that? Everybody has got different ideas what those words mean. So…there’s no point using them really.* (P10, Community Psychiatric Nurse)  *…often I do not find…risk assessment tabs actually to be an assessment of risk. They are more a historical record of risk events which actually is not terrible, because, at least then…I can use that to inform my thinking… but I think to call it a risk assessment is probably…overstating it a little bit. It is actually a record of risk events.* (P05, Psychiatrist)  *…I think if I’m worried about someone and I think their risk…is high…I might use…‘high’. I might…go back to that terminology. I’m not worried about…saying that someone’s high risk if I think that’s the case.* (P15, Psychiatrist) |
|  | 1. Response to violence risk concerns | *You know, you might have service users who don’t want you to disclose anything to family members, but I think…risk trumps everything, really…if you can’t get them on board, then you would have to make that decision whether you breach confidentiality, but if it’s about minimising the risk I think you…have to do that.* (P18, Social Worker) |
| Challenges and barriers to assessing violence risk | 1. Lack of established patterns | *…we haven’t got this huge backlog of information…and so we haven’t got a real picture of someone’s pattern of behaviour, whether that’s…when they’re well and just their lifestyle and how they interact with people or in the context of when they’re unwell*… (P16, Occupational Therapist) |
|  | 1. Stigma, sensitivity and engagement | *I think…we have talked an awful lot in mental health about collaborative risk assessment, but I actually think it’s something that clinicians…find quite difficult sometimes, to talk about risks in a very…pragmatic and involving way.* (P17, Community Psychiatric Nurse).  *I feel a kind of hesitation to ask about it* [violence risk]*…unless there’s an obvious flag, in case it…offends somebody…And I’m…inferring that perhaps, because they’ve got psychosis, they must be violent… I’m really…wary of reinforcing or indicating that I believe the stereotype that’s in the media that people with psychosis are dangerous…We see young people…and it feels relevant to keep things low key and not to make them feel labelled early on.* (P09, Psychologist)  *… you can very easily imagine a situation where the patient is reluctantly seeing you in clinic, has no interest in engaging with you and there is no way of realistically just broaching it saying, “Well, your parents said that you have been violent towards them, what is going on?”…it is very hard to do that.* (P05, Psychiatrist) |
|  | 1. Non-disclosure and access to conviction history | *… people are worried about the consequences, like does this mean that I’m gonna suddenly be rushed into hospital? Are you gonna just suddenly phone the police? And you’re trying to explain that often we gather information, just to gather a picture and how to best support them, but I think there’s some…reluctance like…do you need to know everything about me? And maybe then not seeing the relevance, why do you need to know I’m on probation? (P16, Occupational Therapist)*  *I wonder if there should be a clearer process for getting information, and if it should be more consistent, like how much information is appropriate to share between the agencies…from my experience it doesn’t seem to be something that’s particularly consistent. (P12, Community Psychiatric Nurse)*  *…one thing that I have found frustrating in the past is when you are told that there are problems relating to actual forensic history. But you are not privileged to look at the actual police records. And I know that is a tricky issue but just purely selfishly from my perspective it is frustrating that I know there is risk that you are not party to that we effectively manage*. (P05, Psychiatrist) |
|  | 1. Subjectivity of clinical judgement | *…you’re sort of just guessing it and running it past other people on your instincts and beliefs and bits of knowledge…* (P02, Community Psychiatric Nurse) |
|  | 1. Handover and inter-agency communication | *Clearly…when we’re working with people and for example if they become unstable…we might need to look at additional care…you’d have to be quite clear about the risk to a team that hadn’t had any involvement with them. So, where…violence or aggression…is a potential, you need to be able to… relay that… (P07, Social Worker)* |
|  | 1. Time pressure | *I’ll hold my hands up, the…times that I haven’t updated the risk assessment when probably I should have done, but…it’s the time factor…that quite often gets in the way. (P03, Occupational Therapist)* |
| General attitudes to violence risk | 1. Clinical importance | *It’s important* [violence risk] *because in psychosis… you can lose insight…and believe you were in a situation where…you’re threatened and you need to protect yourself. It’s quite common for people to use weapons when they’re feeling persecuted. Or to hold weapons…like a knife under the pillow or they go around with. That’s not uncommon at all.* (P01, Psychiatrist)  *…it’s really quite important to be addressing it* [violence] *and ensuring that that risk is assessed and managed. I’d say that’s probably the main thing after suicide that I…worry about as a clinician...* (P12, Community Psychiatric Nurse) |
|  | 1. Violence as an “other” risk | *To be completely honest with you, we have had nothing on violence. I think historically, certainly within mental health, it has always been about the risk of suicide that we…tend to…look for primarily…we had a full day training in identifying risk factor for suicides… but…very, very little in terms of violence to others…* (P14, Other Allied MH professional)  *I think violence seems, at least in my training anyway it never really came up….* [there was a] *lot of emphasis on self-harm and suicide but I do not feel there is anything I can lean on in the same way for risk of violence.* (P02, Community Psychiatric Nurse) |
|  | 1. Remit of mental health services | *…just because somebody has mental health issues does not mean that they can’t be aggressive and violent, which I can’t do anything about in the sense that that’s a police matter… who does what and taking responsibility sometimes can be difficult… (P14, Other Allied MH Professional)*  *You need to assess it [violence risk]…accurately…But you don’t want to focus on it too much… we’re here to try and help somebody get better from an illness…and our main…priority is to…get people back on their feet and improve their quality of life… keeping other people safe is…essential but…our main focus is not risk management. (P15, Psychiatrist)* |
|  | 1. Variations in confidence and experience | *…I know there’s bound to be things like…age groups…demographics and things…that make the risk of violence greater, aren’t there?...So maybe if I did know…static risk factors, if I did know that information then maybe my confidence would increase.* (P10, Community Psychiatric Nurse)  *Not at all* [comfortable with assessing violence risk]. *I feel a little bit like it is alchemy…I am looking at all the available information I have and then I am not necessarily sure I am systematically thinking about the relevant risk factors, because I do not know the literature and I do not work in forensics...* (P05, Psychiatrist) |
|  | 1. Individual clinician culpability and the clinical team | *…there’s always that dreaded thing of if something goes wrong…is what you’ve done legally defensible…that’s sort of one of the reasons why…I might run things past…other colleagues…* (P08, Social Worker) |

**Appendix 4**

Table of themes and exemplar quotes from patient and carer interviews.

| **Themes** | **Exemplar Quotes** |
| --- | --- |
| Acceptability of broaching topic of violence risk | *…I think for the safety of people and the staff, they should definitely check* [about violence]*. There’s no stereotypes about it, you know, if you go into a mental hospital…some staff will have past experience of violence…like I say you can never tell with people, so…I think you should always…be sure who you’re dealing with.* (P8, Patient)  *They definitely asked about have I been in trouble with the police…I do remember them talking about that in the interview when I first went to* [the assessment]*, and then it was brought up…just as they were trying to establish everything that had happened and home stuff…I was…forthcoming with my answers to all of them, because I just needed help basically…I knew that I was being supported...* (P12, Patient) |
| Language and framing of violence risk assessment | *I think…just being…open and there is nothing wrong with a question that’s…to the point, really. I think it’s better to be crystal clear and ask the question rather than faff around and not really ask the question clearly…and then making it clear that they do this with everybody, rather than it’s, you know, “We picked on you because we think you’re a risk”...* (P5, Patient)  *It was surprising* [to be asked about violence history] *because I was expecting to get asked about my medical...my health history and then all of a sudden I was asked about…crimes…if you just explain it…why they’re asking us…I think that should be enough.* (P4, Patient) |
| Seeking collateral history for risk assessment | *…they're…just…checking to see if you've got any…criminal past…because they* [patient] *might have been violent to somebody, and…they* [clinicians] *just wanna check…what's happened…it's all in one thing because the health authority, the police…they've got to know what's been happening in a person's past to…understand what's been going on.* (P1, Patient)  *You may not feel able to disclose it in front of the person, but if* [clinicians] *can find that information out some other way it’s got to be helpful for managing the risk to the family…in cases of domestic violence it is often very difficult…to disclose everything in front of the person. There may be details that haven’t been shared and to get that information is really helpful.* (P9, Carer)  *…if you’re living very closely with someone…we’re not experienced professionals in terms of their symptoms or their problems, but…we know them generally speaking as carers…we know them better than…whoever is treating them does. So, whilst we might not understand the problems, I don’t think that a full picture can necessarily be gained without more input from close family members…* (P2, Carer) |
